# Decreased eukaryotic initiation factors expression upon temozolomide treatment—potential novel implications for eIFs in glioma therapy

**DOI:** 10.1007/s11060-023-04451-y

**Published:** 2023-10-31

**Authors:** Stefanie Krassnig, Stefan L. Leber, Andrea Orthmann, Nicole Golob-Schwarzl, Heinrich Johann Huber, Christina Wohlrab, Christina Skofler, Mirjam Pennauer, Andrea Raicht, Anna Maria Birkl-Toeglhofer, Michael Naumann, Kariem Mahdy-Ali, Gord von Campe, Marlene Leoni, Joshua Alcaniz, Jens Hoffmann, Thomas Wälchli, Serge Weis, Martin Benesch, Johannes Haybaeck

**Affiliations:** 1 Diagnostic & Research Center for Molecular BioMedicine, Department of Neuropathology, Institute of Pathology, Medical University of Graz, Graz, Austria; 2 EPO Berlin Buch GmbH, Berlin, Germany; 3 Center for Biomarker Research in Medicine, Graz, Austria; 4 Drug Discovery Sciences, Dr. Boehringer Gasse 5-11 A-1121, Boehringer Ingelheim RCV GmbH & Co KG, Vienna, Austria; 5 Department of Pathology, Medical University of Innsbruck, Müllerstraße 44, Innsbruck, 6020 Austria; 6 Division of Paediatric Haematology and Oncology, Department of Paediatrics and Adolescent Medicine, Medical University of Graz, Graz, Austria; 7 Department of Neurosurgery, Medical University of Graz, Graz, Austria; 8 Division of Neuropathology, Kepler University Hospital, Johannes Kepler University, Neuromed Campus, Linz, Austria; 9 Department of Pathology, Medical Faculty, Otto-von-Guericke University Magdeburg, Magdeburg, Germany; 10 German Center for Neurodegenerative Diseases (DZNE), Magdeburg, Germany; 11 Division of Neuroradiology, Vascular & Interventional Radiology, Department of Radiology, Medical University of Graz, Auenbruggerplatz 9, Graz, 8036 Austria; 12 Group of CNS Angiogenesis and Neurovascular Link, Neuroscience Center Zurich, Division of Neurosurgery, University and University Hospital Zurich, Zurich, Switzerland; 13 Division of Neurosurgery, University Hospital Zurich, Zurich, Switzerland; 14 Group Brain Vasculature and Perivascular Niche, Division of Experimental and Translational Neuroscience, Krembil Brain Institute, Toronto Western Hospital, University Health Network, Krembil Research Institute, University of Toronto, Toronto, ON Canada; 15 Division of Neurosurgery, Department of Surgery, Toronto Western Hospital, University of Toronto, Toronto, ON Canada; 16 Institute of Experimental Internal Medicine, Otto Von Guericke University, Magdeburg, Germany

**Keywords:** Astrocytoma, Glioma, Eukaryotic initiation factors, Temozolomide, Glioma therapy

## Abstract

**Purpose:**

Since glioma therapy is currently still limited until today, new treatment options for this heterogeneous group of tumours are of great interest. Eukaryotic initiation factors (eIFs) are altered in various cancer entities, including gliomas. The purpose of our study was to evaluate the potential of eIFs as novel targets in glioma treatment.

**Methods:**

We evaluated eIF protein expression and regulation in 22 glioblastoma patient-derived xenografts (GBM PDX) after treatment with established cytostatics and with regards to mutation profile analyses of GBM PDX.

**Results:**

We observed decreased expression of several eIFs upon temozolomide (TMZ) treatment independent from the phosphatidylinositol 3-kinase (PI3K)/ AKT/ mammalian target of the rapamycin (mTOR) signalling pathway. These effects of TMZ treatment were not present in TMZ-resistant PDX. Combination therapy of regorafenib and TMZ re- established the eIF/AKT/mTOR axis.

**Conclusion:**

Our study provides novel insights into chemotherapeutic effects on eIF regulation in gliomas and suggests that eIFs are interesting candidates for future research to improve glioma therapy.

**Supplementary Information:**

The online version contains supplementary material available at 10.1007/s11060-023-04451-y.

## Introduction

Gliomas represent a heterogeneous group of brain tumours originating from glial cells. They are classified based on their phenotype as well as genotype according to the 2021 WHO classification [1]. Treatment strategies for gliomas comprise surgical resection and radio-chemotherapy [2]. However, recurrences are frequent in this kind of tumour due to diffuse and infiltrative growth and the overall outcome is still considered poor [2–5]. In the ongoing efforts for a more effective glioma therapy, targeted treatment modalities might be promising tools to provide new treatment options.

eIFs are crucial for the translation from mRNA into proteins at the initiation step [6]. Because an aberrant regulation of the translation initiation step leads to an abnormal gene expression, eIFs seem to be involved in suppression as well as promotion of carcinogenesis [7]. In fact, altered eIF signalling has been reported in vitro [8–11] and in vivo [12–14] in several tumour entities including gliomas [7, 15]. eIF- signalling is mainly regulated by the eIF4F-complex via either the PI3K/AKT/mTOR or the RAS/RAF/MAPK pathways. In PI3K/AKT/mTOR-dependent activation, phosphorylation of eIF- 4E-binding protein 1 (4E-BP1) is facilitated by mTOR. This leads to the dissociation of BP1 and eIF4E and allows the formation of the eIF4F-complex [16]. The RAS/RAF/MAPK pathway activation increases the translation of selective mRNAs through phosphorylation of eIF4E by mitogen-activated protein kinase (MAPK)- interacting serine/threonine kinases 1 and 2 [17]. While the precise role of the RAS/RAF/MAPK pathway in glioblastomas is still the subject of research, the PI3K/AKT/mTOR pathway in glioblastomas has been studied in detail [18]. In this study, we comprehensively characterized eIF expression in 22 GBM PDX upon therapeutic intervention. The aim of the study was to evaluate the potential of eIFs as targets in the development of new glioma therapy options.

## Material & methods

### Patient samples

The study was reviewed and approved by the institutional ethics committee of the Medical University of Graz (MUG) according to Austrian and European laws (24–402 ex 11/12). As controls for biochemical and immunohistochemical analysis, non-neoplastic (“healthy”) cortical control brain tissue (CCBT) was collected post-mortem at the Department of Pathology of the MUG (biochemical analyses: n = 12, immunohistochemistry: n = 15). Astrocytoma samples for biochemical (pilocytic astrocytoma WHO grade I: n = 10, diffuse astrocytoma WHO grade II: n = 13, anaplastic astrocytoma WHO grade III: n = 8, GBM WHO grade IV: n = 13) and immunohistochemical analyses (pilocytic astrocytoma WHO grade I: n = 19, diffuse astrocytoma WHO grade II: n = 24, anaplastic astrocytoma WHO grade III: n = 21, GBM WHO grade IV: n = 20) were obtained from the Biobank of the MUG and the Brain Biobank of the Division of Neuropathology, Neuromed Campus, Kepler University Linz. All samples were neuropathologically classified by board-certified neuropathologists according to the 2016 WHO classification (J.H. and S.W.). Information about neuropathological classification, age, gender, and IDH1 mutation status was recorded for the immunohistochemical analyses (Table S3).

For the establishment of PDX, GBM samples from patients, who had given their informed consent before surgery, were acquired at the Department of Neurosurgery of the MUG. Immediately after surgical resection the tumour samples were placed into RPMI Medium 1640 containing 100 µg/ml gentamicin (Thermo Fisher Scientific, Waltham, MA, US) under sterile conditions and transported without delay to the animal facility overnight (EPO Berlin-Buch GmbH, Berlin, Germany). Information on mutation status (Illumina® TruSeq Amplicon-Cancer Panel) and neuropathological parameters (GFAP, Ki67/MIB2, MAP2, MGMT methylation, IDH1 status) was documented (Table S1).

### Tissue processing for biochemical analyses

Frozen tissue samples were homogenized in NP-40 lysis buffer (0.05 M Tris-HCl, 5 mM NaCl, 0.5% NP-40, 0.1 mM Pefabloc*®*, 1 mM DTT*)* supplemented with cOmplete™ Protease Inhibitor Cocktail and PhosSTOP™ phosphatase inhibitor using the MagNA Lyser homogenizer (all Roche Diagnostics, Risch-Rotkreuz, Switzerland). Protein concentration was determined using the Bradford protein assay (Bio-Rad Laboratories GmbH, Munich, Germany). NP-40 lysates were used for protein and mRNA analyses.

### Immunoblot analysis

Immunoblot analysis was performed as described previously [19]. Briefly, total protein lysates (30 µg/sample) were used for SDS-gel electrophoresis. Primary antibodies were incubated in 5% bovine serum albumin (BSA) in TBS-T (antibody dilutions listed in Table S2) over night at 4 °C. Incubation with a horseradish peroxidase conjugated secondary antibody (dilutions: anti-mouse 1:3000, anti-rabbit 1:5000; GE Healthcare Life Sciences, Buckinghamshire, England) was carried out for 1 h at room temperature. Semi-quantitative evaluation of immunoblots was performed using the ImageJ software (NIH, MD, United States, 20). Relative densities were calculated by normalizing density values for each protein to the GAPDH loading control. To evaluate the changes in protein expression in GBM PDX upon chemosensitivity testing, relative densities were normalized to the PBS control to calculate the x-fold change.

### Establishment of GBM PDX

All animal experiments were performed in accordance with the Guidelines for the Welfare and Use of Animals in Cancer Research [21] and according to the German Animal Protection Law, approved by the local authorities. Surgical tumour samples were cut into pieces of 3–4 mm and immediately transplanted subcutaneously into 2 to 4 immunodeficient NOD/SCID mice (Taconic, Lille Skensved, Denmark). Mice were observed daily for tumour growth. At a size of about 1 cm^3^, tumours were removed and passaged to naive NMRI:nu/nu mice (Janvier Labs, Le Genest-Saint-Isle, France). After three successful passages with NMRI:nu/nu mice, further in vivo studies (testing for chemosensitivity) were initiated.

### Chemosensitivity testings in GBM PDX

The chemotherapeutic response of the passagable tumours was determined in female NMRI:nu/nu mice (Janvier Labs). For that purpose, one tumour fragment was transplanted s.c. to a defined number of mice. Once tumours became palpable, tumour size was measured twice weekly with a calliper-like instrument. Individual tumour volumes (V) were calculated by the formula V= (length x width^2^)/2 and related to the values at the first day of treatment (relative tumour volume, RTV). Median treated to control (T/C) values of RTV was used for the evaluation of each treatment modality. The body weight of mice was determined every 3 to 4 days, and the change in body weight was taken as variable for tolerability.

When mean tumour volume reached the indicated starting volume (80–120 mm^3^), the mice were randomized to six treatment arms (five mice per group) for single treatment studies (treatment panel 1). In the combination treatment studies, the mice were randomized to seven treatment arms (five mice per group; treatment panel 2). Mice were treated in single studies using the drug dosages and treatment schedules listed in Table S3. Control mice in both study types were orally treated with the vehicle alone (phosphate buffered saline (PBS)). Doses and schedules were chosen according to previous experience in animal experiments and represent the maximum tolerated or efficient doses. The injection volume was 0.2 ml/20 g body weight. At the end of the experiments, tumours were excised, immediately snap frozen and stored at − 80 °C for further analyses.

### Mutation profile analysis of GBM PDX

Genomic DNA was isolated from PDX samples using the QIAamp DNA Mini Kit (Qiagen, Hilden, Germany) according to the manufacturer’s instructions. DNA was quantified spectrophotometrically using the NanoDrop ND-100 (NanoDrop Technologies, Inc., Wilmington, DE, US). Mutation analysis was performed on an Illumina MiSeq Platform (Illumina, San Diego, CA, US) using the Illumina® TruSeq Amplicon-Cancer Panel covering 212 target regions in 48 cancer-related genes. Somatic sequence variations were determined by the Illumina Variant Caller as provided by the MiSeq analysis workflow. Only sequence variations with a frequency above 15% were considered.

### Statistical analysis

Pearson correlation analysis for the relative densities of immunoblots from treated GBM PDX was performed in R 3.3.0 (R Development Core Team, 2008) using the method rcorr of package hmisc (Harrell FE Jr. Package ‘HMISC’. Updated June 4, 2010; available at: https://cran.r-project.org/web/packages/Hmisc/index.html. Accessed June 17, 2017) whereby absolute Pearson correlation coefficient values > 0.7 were considered as threshold for a strong link and such between 0.5 and 0.7 as indicative.

Statistical analyses and graphs were generated using Graph Pad Prism 4.03 (GraphPad Software Inc., CA, US). All data were tested for normal distribution using the Kolmogorov-Smirnoff test. For evaluation of biochemical and immunohistochemical data, one-way analysis of variance (ANOVA) was used followed by Bonferroni’s multiple comparison test (normally distributed data) or Kruskal-Wallis test followed by Dunn’s multiple comparison test (not normally distributed data) to analyse the eIF expression either in astrocytomas WHO grade I-V compared to control tissue or in chemosensitivity testings in GBM PDX. Significance levels were set at p < 0.05.

For Spearman correlation analysis between eIF expression and tumour response after TMZ treatment we built the treatment to control ratio in percent (T/C%). This resulted in a response rate considering a T/C% of > 50% a non-responder, T/C% of 50–35% as a minimal responder, 35–20% as a weak responder, 20 − 5% as a moderate responder and less than 5% as a strong responder (Table S4).

### Role of the funding source

Besides funding, there was no involvement of the funding source in the preparation of the manuscript.

## Results

### TMZ treatment decreased eIF protein expression in GBM PDX

**Fig. 1:**
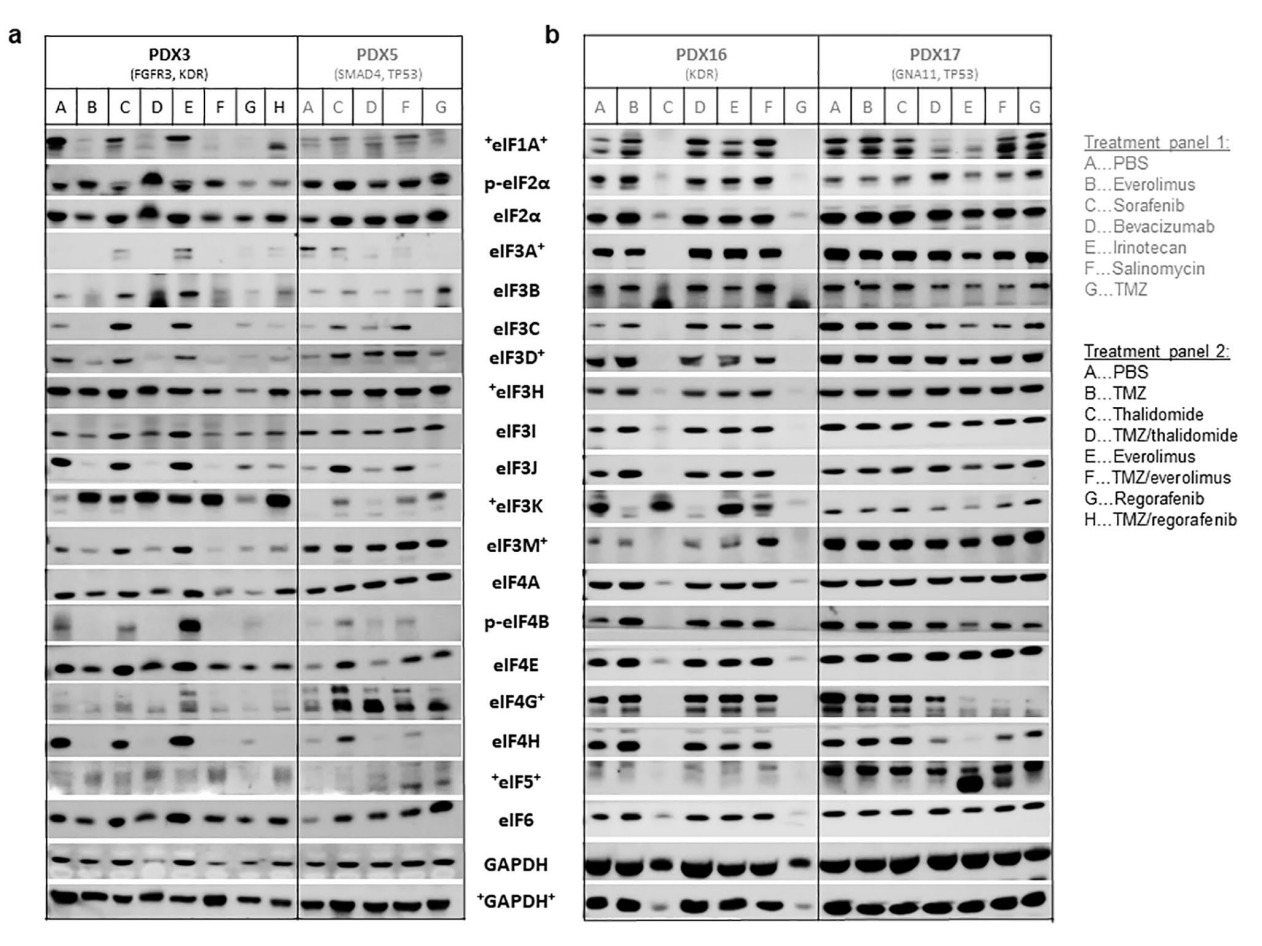
Altered eIF protein expressions after chemosensitivity testing in GBM PDX. Representative immunoblots for eIF protein expression in PDX after chemosensitivity testing. (A) PDX5, (B) PDX16 and PDX17 received single drugs (treatment panel 1), whereas PDX3 also received drug combinations (treatment panel 2). Treatment panel 1 (PDX5, PDX16, PDX17): PBS-control, Everolimus (EVE), Sorafenib (SOR), Bevacizumab (BEV), Irinotecan (IRI), Salinomycin (SAL) and temozolomide (TMZ). Treatment panel 2 (PDX3): PBS-control, temozolomide (TMZ), Thalidomide (THA), TMZ/Thalidomide (TMZ + THA), Everolimus (EVE), TMZ/Everolimus (TMZ + EVE), Regorafenib (REG) and TMZ/Regorafenib (TMZ + REG). (**A**) PDX3 (*FGFR3* and *KDR* mutation) and (B) PDX16 (*KDR* mutation) were not resistant to TMZ, whereas (A) PDX5 (*SMAD4* and *TP53* mutation) and (**B**) PDX17 (*GNA11* and *TP53* mutation) revealed TMZ-resistance. eIFs marked with a “+” were normalized to the + GAPDH + loading control (12.5% SDS gel), whereas eIFs without any mark were normalized to GAPDH (8% SDS gel). *Abbreviations: eIF: Eukaryotic initiation factor; GAPDH: Glyceraldehyde 3-phosphate dehydrogenase; PBS: Phosphate buffered saline; PDX: Patient-derived xenograft*

**Fig. 2:**
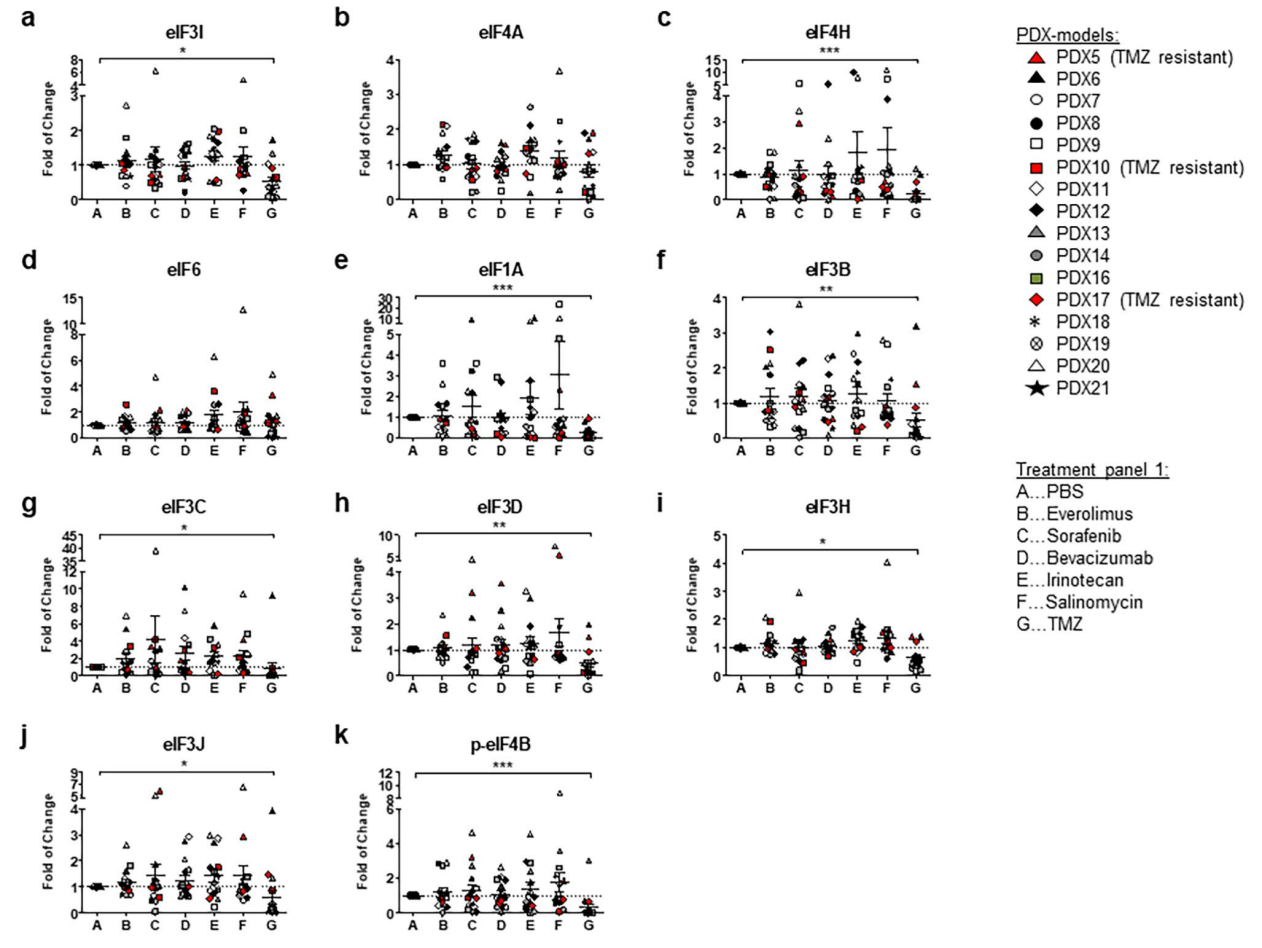
Decreased eIF protein expression in GBM PDX upon TMZ treatment in densitometric immunoblot analyses. We analyzed the effect of everolimus, sorafenib, bevacizumab, irinotecan, salinomycin and temozolomide (TMZ) on eIF protein expression compared to a PBS control group using immunoblot analysis. We investigated 16 different PDX. Except for PDX5, PDX10 and PDX17 (marked in red), TMZ reduced tumour growth in all PDX. For relative densities, expression of (**A**) eIF3I, (**B**) eIF4A, (**C**) eIF4H, (**D**) eIF6, (**E**) eIF1A, (**F**) eIF3B, (**G**) eIF3C, (**H**) eIF3D, (**I**) eIF3H, (J) eIF3J and (K) p-eIF4B was normalized to the loading control (GAPDH). We normalized relative densities to the PBS control to calculate the x-fold change (Scatter dot blot + SEM). Numbers: n = 16/treatment. Statistical analysis: 1-way ANOVA with Bonferroni posttest. Significance levels: *p < 0.05; **p < 0.01, ***p < 0.001. *Abbreviations: eIF: Eukaryotic initiation factor; GAPDH: Glyceraldehyde 3-phosphate dehydrogenase; PBS: Phosphate buffered saline; PDX: Patient-derived xenograft; TMZ: Temozolomide, EVE: Everolimus, SOR: Sorafenib, BEV: Bevacizumab, IRI: Irinotecan, SAL: Salinomycin*

To investigate whether established cytostatics influence eIF protein expression, 22 GBM PDX were analyzed upon chemosensitivity testing. TMZ most efficiently reduced tumour growth in almost all PDX compared to PBS-treated controls, except for PDX5, PDX10 and PDX17. The second most effective agent reducing tumour growth was irinotecan. Bevacizumab reduced tumour growth in 50% of the PDX, but was not as effective as TMZ or irinotecan (Figure S1 A). Protein expression of eIFs was not significantly affected by the investigated treatment panel 1 (everolimus, sorafenib, bevacizumab, irinotecan, salinomycin, TMZ), except for TMZ (Figs. 1 and 2). Protein levels of most eIFs were significantly decreased in tumours after TMZ treatment compared to PBS-treated controls. eIF3I (p < 0.05; Fig. 2A) and eIF4H (p < 0.001; Fig. 2C) were significantly down-regulated upon TMZ treatment. Relative densities of eIF3I were reduced up to 90% upon TMZ treatment compared to PBS treated controls (mean relative density reduction of 63% ± 26%, non-responders were excluded). For eIF4H, relative densities were reduced even up to 100% upon TMZ treatment compared to PBS treated (mean relative density reduction of 90% ± 19%, non-responders were excluded). However, reduced eIF protein expression was not observed in the TMZ-resistant PDX5 and PDX17 (Figs. 1A and B and 2). eIF4A revealed differential results as protein expression was either partly reduced or showed no alterations (Fig. 2B). eIF6 protein expression did not respond to TMZ treatment (Fig. 2D). eIF1A (p < 0.001), eIF3B (p < 0.01), eIF3C (p < 0.05), eIF3D (p < 0.01), eIF3H (p < 0.05), eIF3J (p < 0.05) and p-eIF4B (p < 0.001; Fig. 2E-K) were also significantly decreased upon TMZ treatment. Other eIFs revealed no significant alterations upon treatment (Figure S2, panel1). In treatment panel 2, TMZ also most efficiently reduced tumour growth except for PDX14 and PDX20 (Figure S1 B). Regarding tumour growth and eIF expression, no additional effect was observed when combining TMZ with thalidomide, everolimus or regorafenib (Fig. 1B). Again, TMZ reduced eIF protein expression less efficiently in TMZ-resistant PDX. Results for the combination treatments were not statistically significant, due to the low number of PDX (Figure S3).

The correlation analysis revealed a significant negative correlation of eIF3I with the scoring for the response rate (p = 0.015, correlation coefficient − 0.593), indicating decreasing eIF3I expression in better responders. We observed statistical trends for eIF2alpha, eIF3H and eIF6, however there were no statistically significant correlations for the remaining eIFs (Table S5).

### PI3K/AKT/mTOR-independent downregulation of eIFs upon TMZ treated GBM PDX

To identify potential regulatory impact of the PI3K/AKT/mTOR on eIF expression upon chemosensitivity testing, members of this signalling were investigated at the protein level in the treated GBM PDX (Figure S4). A statistically significant decrease was found only for p-p70S6K upon TMZ treatment (p < 0.05; Figure S4E, panel 1).

**Fig. 3:**
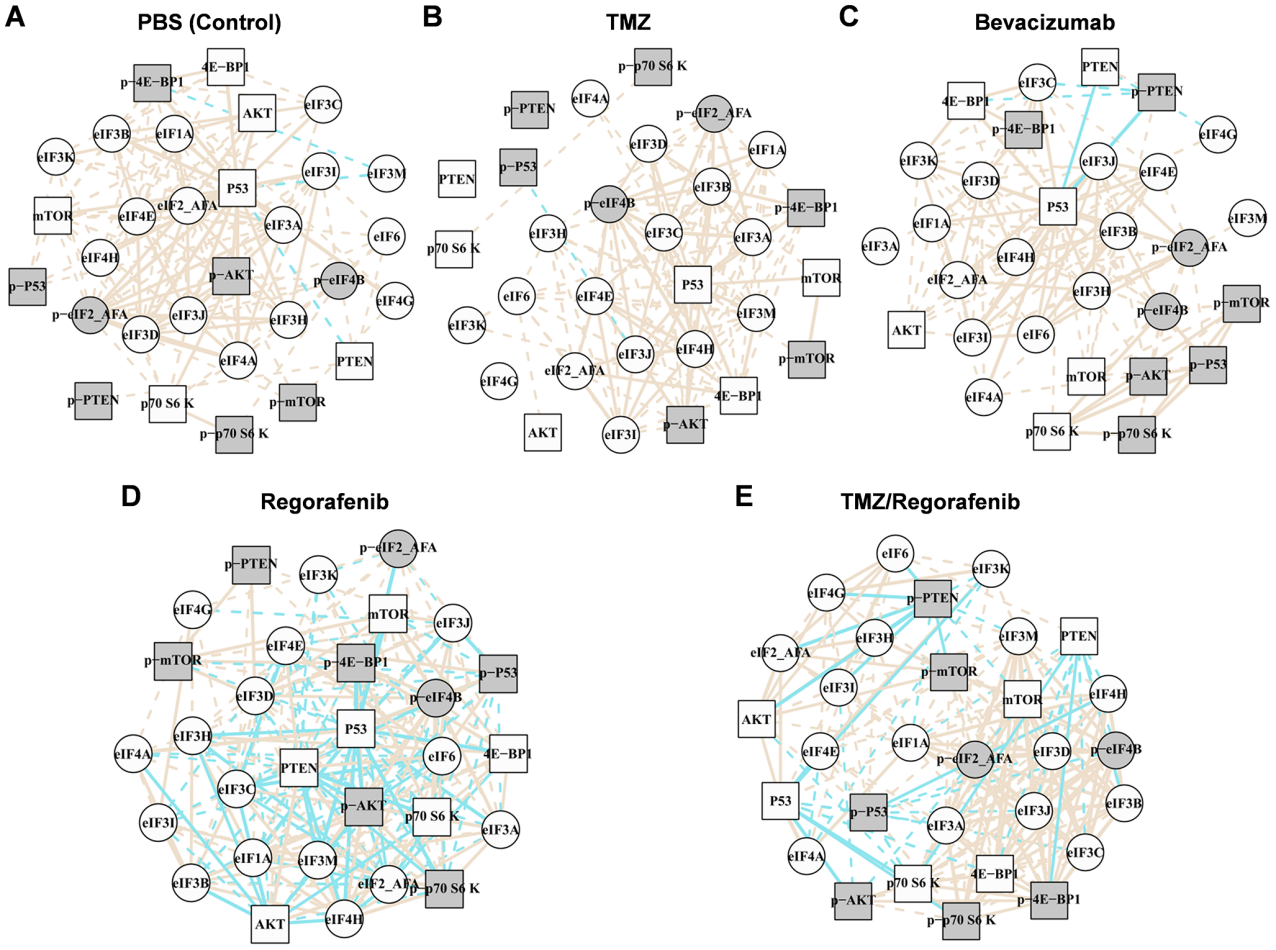
Decreased eIF expression upon TMZ treatment occurs in a PI3K/AKT/mTOR-independent manner. We performed Pearson correlation analyses for key protein expressions to infer regulation patterns from their co-expressions between distinct xenografts. We obtained protein levels from densitometric analyses of immunoblots of GBM PDX. Pictured are correlation analyses for (**A**) PBS control, (**B**) temozolomide (TMZ), (**C**) bevacizumab, (**D**) regorafenib and (**E**) TMZ/regorafenib treatment groups. Boxes represent PI3K/AKT/mTOR signalling members, circles represent eIF signalling members. Grey highlighting indicates Phospho-proteins, beige lines indicate positive correlation coefficients and blue lines indicate negative correlations between two proteins. Line thickness represents the strength of the correlation between two proteins; thick lines are strong correlations whereas dotted lines are weak correlations. Numbers: PBS n = 22, TMZ n = 22, bevacizumab n = 16, regorafenib n = 6, TMZ/regorafenib n = 6. *Abbreviations: 4E-BP1: eIF4E-binding protein 1; AKT: Protein kinase B; eIF: Eukaryotic initiation factor; mTOR: mammalian/mechanistic Target of Rapamycin; p70S6K: p70 Ribosomal protein S6 kinase; PBS: Phosphate buffered saline; PTEN: Phosphatase and Tensin homologue; TMZ: Temozolomide*

In the next step we researched whether drug exposure against tyrosine-kinase-receptor-induced signalling alters PI3K/AKT/mTOR expression. Therefore, a pairwise correlation of the PI3K/AKT/mTOR pathway members was performed in GBM PDX upon PBS (control), TMZ, bevacizumab, regorafenib and TMZ/ regorafenib treatment (Fig. 3A-E).

PBS-treated PDX exhibited the same deregulation as patient tissues from HGG (high grade glioma), supporting the suitability of GBM PDX for chemosensitivity testing (Fig. 3A vs. Figure S4 and S5). GBM patients as well as PBS-treated GBM PDX revealed a loss of correlation between the PI3K/AKT/pathway members. In detail, this loss of correlation occurred between PTEN/AKT/mTOR, mTOR/p70S6K and p-4E-BP1/p-eIF4B. This indicates deactivation of the regulatory functions in this pathway. A gain of correlation on the other hand suggests a reactivation of the respective pathway. The mechanisms of action of TMZ (Fig. 3B), as well as those of bevacizumab (Fig. 3C), did not involve the PI3K/AKT/mTOR pathway, as no additional negative correlations/activations were detected compared to the PBS control. Thus, downregulation of eIF expression upon TMZ treatment was achieved in a PI3K/AKT/mTOR-independent manner. The loss of correlation indicates that TMZ might alter the pathway in a manner that favours other signalling events which may re-use these proteins for other purposes. Regorafenib seemed to be the only treatment that reduced eIF signalling through deactivation of the PI3K/AKT/mTOR pathway. In a comparison with the PBS control, the number of negative correlations was increased upon regorafenib treatment (Fig. 3A vs. Figure 3D). In particular, interactions between mTOR/p70S6K – p-4E-BP/p-eIF4B could be reconstituted upon regorafenib treatment but it has to be considered that the number of PDX treated with regorafenib is low (n = 6). The combination of TMZ/regorafenib (Fig. 3E) revealed a similar interaction in a less extensive manner, which supports the results. The reduced activation in the combination treatment could be explained by the fact that TMZ alone did not affect the PI3K/AKT/mTOR pathway at all (Fig. 3B vs. Figure 3E).

### Genetic alterations such as TP53 mutations in TMZ- resistant gliomas

Frequently occurring genomic mutations during gliomagenesis might partly explain TMZ-resistance of some GBM PDX and also unchanged eIF protein expression upon TMZ treatment. Therefore, we characterized the mutational status of all investigated GBM PDX. Five GBM PDX in our study were TMZ-resistant as the tumour size did not decrease upon TMZ treatment (treatment panel 1: PDX5, PDX10, PDX17; treatment panel 2: PDX14, PDX20; Figure S1). The mutations most frequently found in these TMZ-resistant models were *TP53* (PDX5, PDX17) and kinase insert domain receptor (*KDR*) mutations (PDX14, PDX20). However, other PDX also revealed mutations of these two genes without developing a TMZ-resistance (*TP53*: PDX12, PDX18; Table S1). Genetic alterations such as *TP53* mutations could lead to the development of TMZ-resistances, but this hypothesis needs to be verified in a higher number of PDX.

## Discussion

Gliomas represent a heterogeneous group of tumours with varying clinical outcomes. Despite huge efforts in the development of new treatment options for gliomas, results are still modest with numerous failed clinical trials [22]. The purpose of our study was to characterize various eIFs in gliomas after chemotherapeutic treatment and to evaluate their potential as novel target candidates in glioma therapy.

Several eIFs have already been demonstrated to be altered in different cancer entities, including gliomas [7]. Upregulation of eIF3B [8] was described in glioma patient samples. eIF3C [9], eIF3D [10], eIF4E and 4E-BP1 [13] correlated with tumour grade with significantly higher levels in HGG compared to LGG (low grade glioma). Our data confirm a statistically significant increase in protein expression of eIF3B, eIF3D, and p-4E-BP1, in particular in HGG. Increased protein and mRNA levels were also found for eIF3C and eIF4E. However, this finding was not statistically significant. In addition to the previously characterized eIFs, we identified elevated expression of a variety of other eIFs in the course of this analysis.

Besides identifying eIFs as possible direct targets for glioma therapy, we performed chemosensitivity testing in 22 GBM PDX to see whether established cytostatics might influence eIF protein expression and if the combination of established cytostatics can enhance the current frontline treatment agent TMZ. In accordance with earlier studies, the alkylating agent TMZ most effectively reduced tumour growth in the investigated PDX [23]. In addition, TMZ significantly reduced the expression of 9 eIFs, including eIF3I and eIFH In a previous study, eIF3I and eIF4H were increased in gliomas and significantly associated with the overall survival of glioma patients [15]. This supports the hypothesis that eIF3I and eIF4H are of interest for future research on the improvement of glioma therapy. Notably, eIF3I but not eIF4H significantly correlated with tumor size reduction after TMZ treatment. This partial lack off correlation might be explained due to involvement of TMZ-resistant PDX.

Surgery, followed by radiotherapy with concomitant TMZ, followed by TMZ chemotherapy is the current standard of care in GBM [2]. Combining TMZ with other agents such as thalidomide, everolimus or regorafenib did not significantly reduce tumour size in comparison to TMZ alone, suggesting no additional effects of combined treatments on tumour size reduction in GBM PDX. Although PDX of GBM overcome some limitations of xenografts from isogenic cell lines, there are still some restrictions such as the resulting tumour size, heterotopic transplantation and their ability to cover only a fraction of the tumour heterogeneity [16].

As combination treatments did not show any additional effect on tumour size reduction, we aimed to investigate whether the combination of cytostatics has beneficial regulatory effects at the molecular level, with a focus on the eIF signalling cascade. eIF signalling is regulated upstream via the PI3K/AKT/mTOR pathway [17] which has previously been demonstrated to be increasingly activated and dysregulated in astrocytomas (WHO grade I-IV) [18, 24]. We found that the regulatory capacity of the AKT/mTOR axis with respect to eIF expression was lost in neoplastic tissue compared to control brain tissue and therefore hypothesized that a re-establishment of the AKT/mTOR axis might have beneficial treatment effects.

Interestingly, down-regulation of eIFs upon TMZ treatment occurred in a PI3K/AKT/mTOR-independent manner. The AKT/mTOR-mediated eIF control was re-established only by regorafenib, while the other examined cytostatics were not able to positively influence this loss. Regorafenib is a multi-tyrosine kinase inhibitor of oncogenic and angiogenic tyrosine receptor kinases [25]. Regorafenib has been tested in rat and human GBM PDX [25, 26] and has been investigated in recurrent GBM in the REGOMA phase II trial (NCT02926222). Re-establishment of the eIF/AKT/mTOR axis through a combination of TMZ and regorafenib might be an interesting new approach for future research as it might improve outcome for GBM patients.

Five of our PDX showed resistance to TMZ and in most of the TMZ-resistant models eIF protein expression also remained stable. Thus, eIF protein expression might reflect a response to TMZ in the investigated GBM PDX. The underlying molecular mechanisms contributing to TMZ-resistance are still poorly understood. High expression of the methylguanine-DNA methyltransferase (MGMT) enzyme, repairing the mutagenic effects of TMZ on DNA, is supposed to be one reason for TMZ resistance [27]. However, additional MGMT-independent mechanisms have also been linked to TMZ resistance [28] and tumour heterogeneity and genetic alterations are assumed to be further reasons for tumour recurrence and treatment failures [29]. The five resistant PDX displayed most frequently *KDR* and *TP53* mutations. Notably, the mutation in *TP53* is known to arise early during gliomagenesis [30] and has previously been shown to increase TMZ-resistance [5]. Increased gene amplification of *KDR*, also known as vascular endothelial growth factor receptor (VEGFR) 2 or Flk-1, was found in GBM [31, 32]. However, the exact role of KDR in TMZ resistance is still under debate.

Our study demonstrates the effects of established cytostatics on eIF expression. Moreover, a combination of TMZ and regorafenib re-established the eIF/AKT/mTOR axis. In line with our previous study [15], we conclude that eIFs, especially eIF3I and eIF4H, are interesting candidates for future glioma therapy research.

### Electronic supplementary material

Below is the link to the electronic supplementary material.

Supplementary Material 1

## Declarations

### Data sharing statement

Original data are stored at the ‘Mendeley Data’ and available in [10.17632/xpkjey7gc3.1] with publication.
