# Supplementary material for: Decreased eukaryotic initiation factors expression upon temozolomide treatment—potential novel implications for eIFs in glioma therapy
Source: J Neurooncol. 2023 Oct 31;165(1):91–100. doi: 10.1007/s11060-023-04451-y (PMC10638187; doi:10.1007/s11060-023-04451-y)
Supplement: Supplementary file 1 — Supplementary Material 1 [file 11060_2023_4451_MOESM1_ESM.docx]

**Supplementary figures with titles and legends**


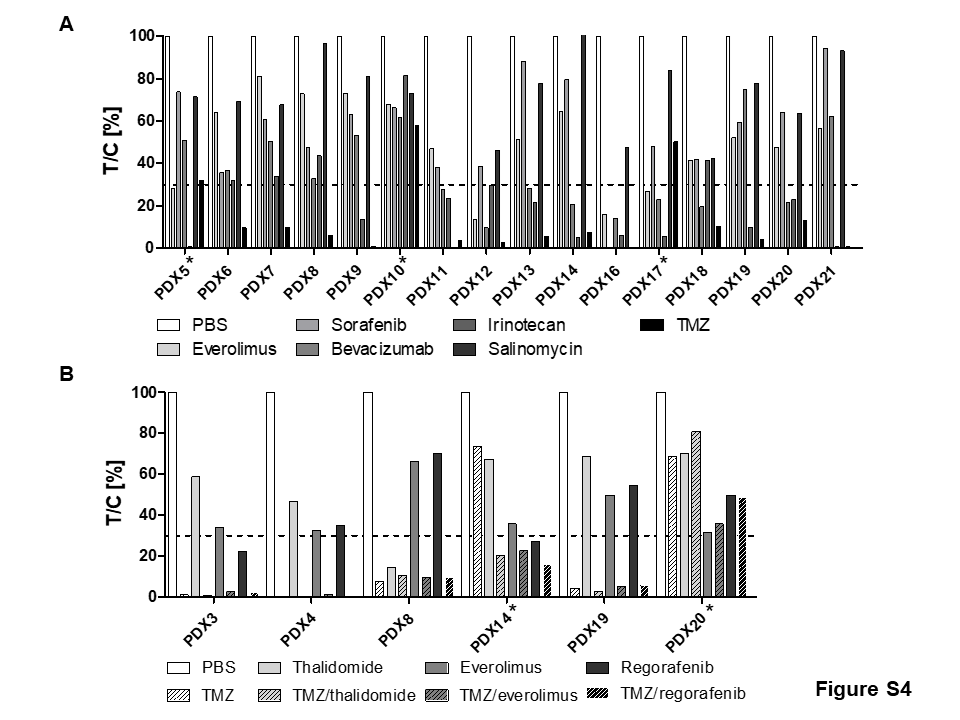


**Figure S1: TMZ reveals the highest antitumour activity in GBM PDX after chemosensitivity testing.** To evaluate drug efficacy in PDX for single treatments (treatment panel 1, A) or drug combinations (treatment panel 1, B), the percentage of treatment-to-control ratio (T/C) was calculated. The T/C values represent the ratio of tumour volume in PBS controls versus the tumour volume of drug-treated PDXs. (A) For treatment panel 1, PDX were treated with PBS (control), sorafenib, irinotecan, temozolomide (TMZ), everolimus, bevacizumab and salinomycin. (B) For treatment panel 2, PDX- were treated with PBS (control), TMZ (B), thalidomide, TMZ/thalidomide, everolimus, TMZ/everolimus, regorafenib and TMZ/regorafenib. The GBM PDX displayed a heterogeneous response to the tested drug panels with strong initial sensitivity to TMZ. Based on a T/C value ≤ 30% as cut-off, 13 PDX were defined as responder and 3 PDX as non-responder to TMZ for treatment panel 1. For treatment panel 2, 4 PDX were defined as responder and 2 PDX as non-responder to TMZ. TMZ-resistant PDX are marked with an asterisk (Treatment panel 1: PDX5*, PDX10* and PDX17*; Treatment panel 2: X14* and X20*). Numbers: Treatment panel 1: n=16; Treatment panel 2: n=16. *Abbreviations: PBS: Phosphate buffered saline; PDX: Patient-derived xenograft; T/C: Treatment-to-control ratio; TMZ: Temozolomide.*


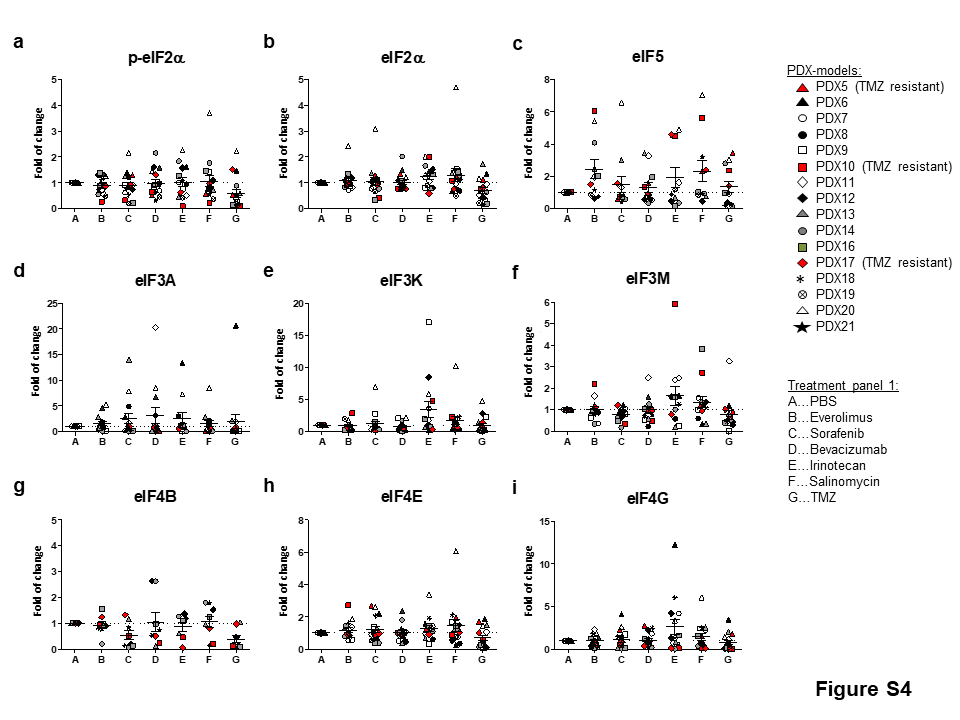


**Figure S2: eIF protein expression after chemosensitivity testing in GBM PDX in densitometric immunoblot analysis (Treatment panel 1).** The effect of everolimus, sorafenib, bevacizumab, irinotecan, salinomycin and temozolomide (TMZ) on eIF protein expression was analyzed in a comparison with the PBS control group using immunoblot analysis. We investigated 16 different PDX, in all PDX, except for PDX5, PDX10 and PDX17 (marked in red), TMZ drastically reduced tumour growth. For relative densities we normalized expression of (A) p-eIF2α, (B) eIF2α, (C) eIF5, (D) eIF3A, (E) eIF3K, (F) eIF3M, (G) eIF4B, (H) eIF4E and (I) eIF4G to the loading control (GAPDH). Then we normalized to the PBS control to calculate the x-fold change. Scatter dot blot + SEM. Numbers: n= 16/treatment. Statistical analysis: 1-way ANOVA with Bonferroni posttest. *Abbreviations: eIF: Eukaryotic initiation factor; PBS: Phosphate buffered saline; PDX: Patient-derived xenograft; TMZ: Temozolomide.*


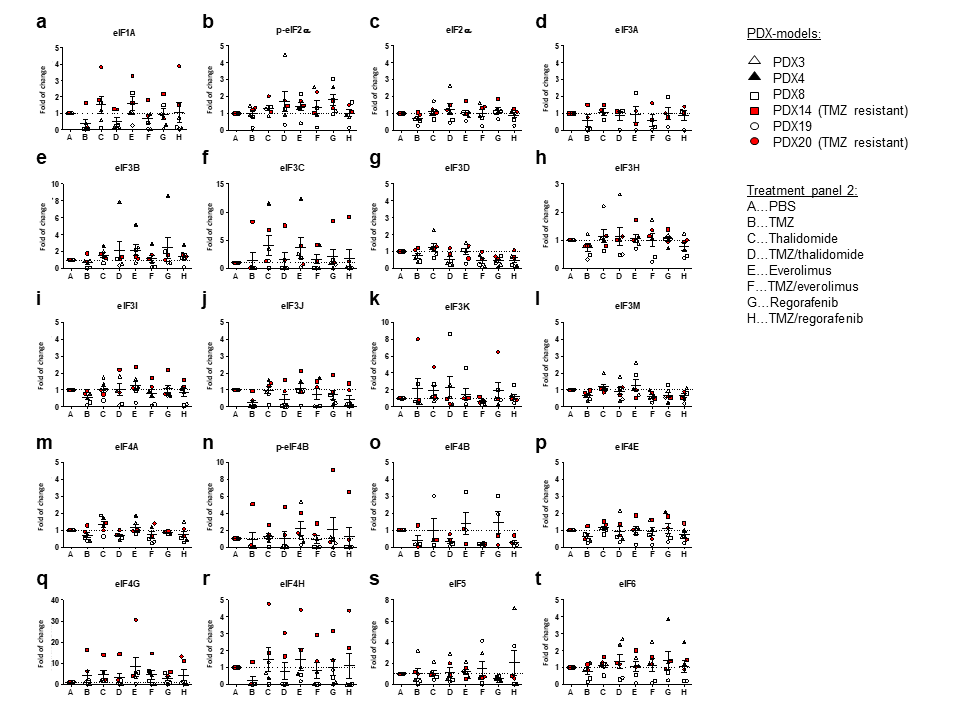


**Figure S3: eIF protein expression after chemosensitivity testing in GBM PDX in densitometric immunoblot analysis (Treatment panel 2).** We analyzed the effect of temozolomide (TMZ), thalidomide, TMZ/thalidomide, everolimus, TMZ/everolimus, regorafenib and TMZ/regorafenib on eIF protein expression in comparison to the PBS control group using immunoblot analysis. We investigated six different PDX. In all PDX except for PDX14 and PDX20 (marked in red), TMZ drastically reduced tumour growth. For relative densities we normalized expression of (A) eIF1A, (B) p-eIF2α, (C) eIF2α, (D) eIF3A, (E) eIF3B, (F) eIF3C, (G) eIF3D, (H) eIF3H, (I) eIF3I, (J) eIF3J, (K) eIF3K, (L) eIF3M, (M) eIF4A, (N) p-eIF4B, (O) eIF4B, (P) eIF4E, (Q) eIF4G, (R) eIF4H, (S) eIF5 and (T) eIF6 to the loading control (GAPDH). Then we normalized relative densities to the PBS control to calculate the x-fold change. Scatter dot blot + SEM. Numbers: n=6/treatment. Statistical analysis: 1-way ANOVA with Bonferroni posttest. *Abbreviations: eIF: Eukaryotic initiation factor; PBS: Phosphate buffered saline; PDX: Patient-derived xenografts; TMZ: Temozolomide.*


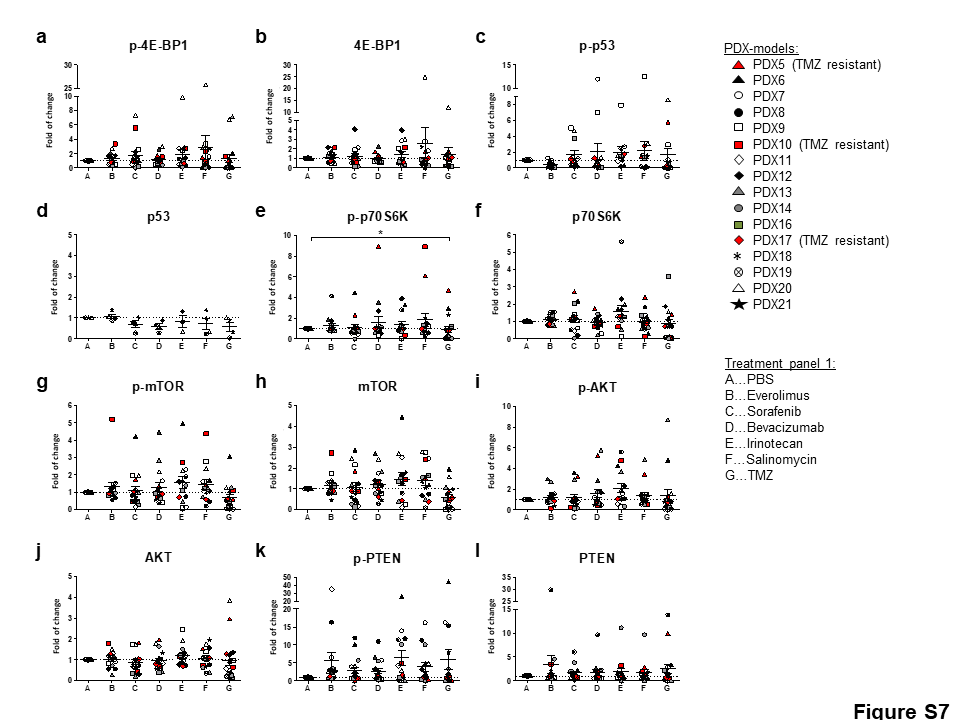


**
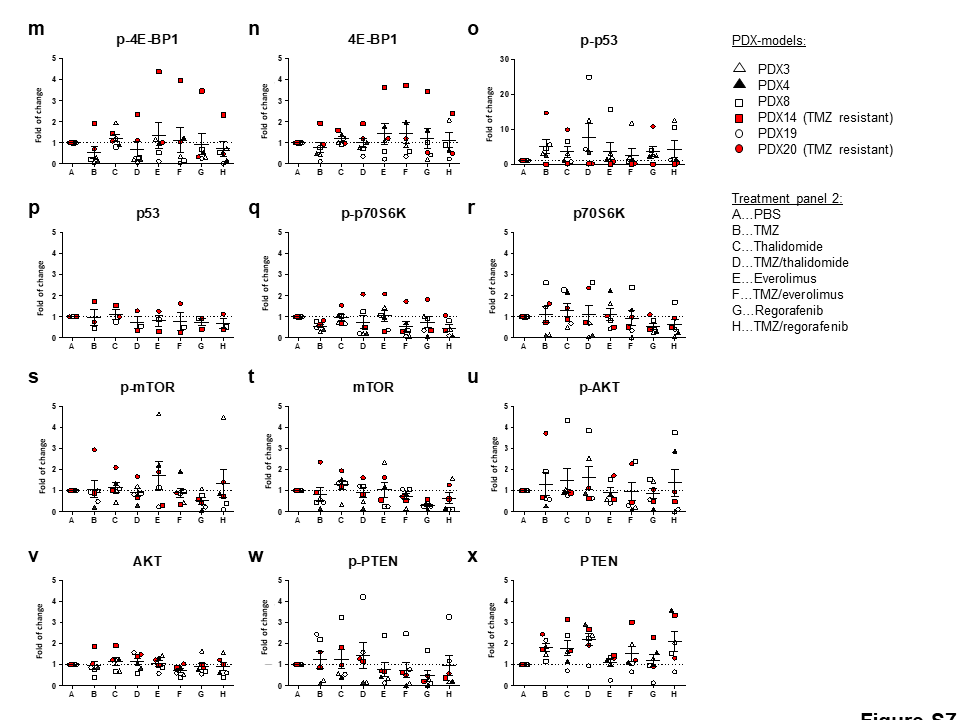
**

**Figure S4: PI3K/AKT/mTOR protein expression after chemosensitivity testing in GBM PDX in densitometric immunoblot analysis.** (A-L) We analyzed the effect of everolimus, sorafenib, bevacizumab, irinotecan, salinomycin and temozolomide (TMZ) on protein expression of members of the PI3K/AKT/mTOR signalling pathway compared to the PBS control group using immunoblot analysis (Treatment panel 1). We investigated 16 different PDX. In all PDX, except for PDX5, PDX10 and PDX17 (marked in red), TMZ drastically reduced tumour growth (M-X). In addition we analyzed the effect of temozolomide (TMZ), thalidomide, TMZ/thalidomide, everolimus, TMZ/everolimus, regorafenib and TMZ/regorafenib on protein expression of members of the PI3K/AKT/mTOR signalling pathway compared to the PBS control group using immunoblot analysis (Treatment panel 2). Six different PDX were investigated. In all PDX, except for PDX14 and PDX20 (marked in red), TMZ drastically reduced tumour growth. For relative densities we normalized, expression of (A, M) p-4E-BP1, (B, N) 4E-BP1, (C, O) p-p53, (D, P) p53, (E, Q) p-p70S6K, (F, R) p70S6K, (G, S) p-mTOR, (H, T) mTOR, (I, U) p-AKT, (J, V) AKT, (K, W) p-PTEN and (L, X) PTEN was normalized to the loading control (GAPDH). We normalized relative densities to the PBS control to calculate the x-fold change. Scatter dot blot + SEM. Numbers: Panel 1: n=16/treatment (p53 n=4/treatment). Panel 2: n=6/treatment (p53 n=3/treatment). Statistical analysis: 1-way ANOVA with Bonferroni post-test. Significance levels: *p < 0.05. *Abbreviations: 4E-BP1: eIF4E-binding protein 1; AKT: Protein kinase B; GAPDH: Glyceraldehyde 3-phosphate dehydrogenase; mTOR: mammalian/mechanistic Target of Rapamycin; p70S6K: p70 Ribosomal protein S6 kinase; PBS: Phosphate buffered saline; PDX: Patient-derived xenograft; PTEN: Phosphatase and Tensin homologue; TMZ: Temozolomide.*

**Supplementary tables with titles and legends**

Table S1: Characterization of GBM PDX used for chemosensitivity testings. For each GBM PDX mutation status (Illumina^®^ TruSeq Amplicon-Cancer Panel) and neuropathological parameters (GFAP, Ki67/MIB2, MAP2, MGMT methylation, IDH1 status) were evaluated. (A) For treatment panel 1, PDX were treated with PBS (control, a), Everolimus (b), Sorafenib (c), Bevacizumab (d), Irinotecan (e), Salinomycin (f) and Temozolomide (TMZ; g). (B) For treatment panel 2, PDX- were treated with PBS (control; a), Temozolomide (TMZ; b), Thalidomide (c), TMZ/Thalidomide (d), Everolimus (e), TMZ/Everolimus (f), Regorafenib (g) and TMZ/Regorafenib (h).Drug efficacy was calculated as the treatment-to-control ratio (T/C) expressed in %. The T/C values represent the ratio of tumour volume in PBS controls versus the tumour volume of drug treated PDXs. PDX were defined as responder with a T/C value < 30% and as non-responder with a T/C value > 30% (marked in grey).

| 1. **Panel 1** | | **Neuropathological Assessment** | | | | | **T/C [%]** | | | | | | |
| --- | --- | --- | --- | --- | --- | --- | --- | --- | --- | --- | --- | --- | --- |
| **Study number** | **Mutations** | **GFAP** | **Ki67/MIB2** | **MAP2** | **MGMT** | **IDH1 (R132H)** | **PBS (a)** | **Everolimus (b)** | **Sorafinib (c)** | **Bevacizumab (d)** | **Irinotecan (e)** | **Salinomycin (f)** | **TMZ (g)** |
| PDX5 | TP53 | negative | 30% | negative | negative | negative | 100.0 | 28.1 | 73.7 | 50.9 | 0.9 | 71.4 | 32.0 |
| PDX6 | PIK3CA, PTPN11 | positive  (100%) | 20% | positive | negative | negative | 100.0 | 63.8 | 35.6 | 36.8 | 32.0 | 69.1 | 9.7 |
| PDX7 | FGRF3,RB1 | positive  (100%) | 80% | negativ | positive (60%) | negative | 100.0 | 81.0 | 60.9 | 50.5 | 34.0 | 67.6 | 9.7 |
| PDX8 | APC, FLT3 | positive  (100%) | 10% | positive | negative | negative | 100.0 | 72.9 | 47.4 | 32.7 | 43.5 | 96.5 | 6.0 |
| PDX9 | ERBB2, GNAQ, KDR,PTEN | positive (40%) | 50% | positive | negative | negative | 100.0 | 73.0 | 62.9 | 53.3 | 13.8 | 80.9 | 0.7 |
| PDX10 | FGRF3, PTEN | positive (70%) | 70% | positive | positive (40%) | negative | 100.0 | 67.9 | 66.3 | 61.7 | 81.3 | 73.2 | 58.1 |
| PDX11 | MET, NRAS, PTEN, RET | positive  (100%) | 80% | positive | negative | negative | 100.0 | 46.9 | 38.0 | 27.7 | 23.5 |  | 3.6 |
| PDX12 | KDR, TP53 | positive  (100%) | 95% | negativ | negative | negative | 100.0 | 13.7 | 38.8 | 9.8 | 29.6 | 46.0 | 2.6 |
| PDX13 | ABL1, ATM, ERBB2,  GNA11, PTEN, VHL | positive  (100%) | 40% | positive | negative | negative | 100.0 | 51.2 | 88.0 | 28.2 | 21.7 | 77.5 | 5.6 |
| PDX14 | KDR, KIT | positive (30%) | 90% | positive | positive (70%) | negative | 100.0 | 64.5 | 79.4 | 20.6 | 5.0 | 100.5 | 7.4 |
| PDX16 | KDR | positive  (100%) | 85% | positive | negative | negative | 100.0 | 16.1 | 0.4 | 14.0 | 6.2 | 47.7 | 0.1 |
| PDX17 | GNA11, TP53 | positive  (100%) | 90% | positive | negative | negative | 100.0 | 26.8 | 47.9 | 22.9 | 5.8 | 83.7 | 50.1 |
| PDX18 | TP53 | positive  (100%) | 90% | positive | negative | negative | 100.0 | 41.6 | 42.0 | 19.5 | 41.3 | 42.2 | 10.2 |
| PDX19 | no mutations | positive  (100%) | 85% | positive | negative | negative | 100.0 | 52.1 | 59.5 | 74.7 | 9.9 | 77.8 | 4.4 |
| PDX20 | KDR, PIK3CA | positive  (100%) | 80% | positive | negative | negative | 100.0 | 47.7 | 64.0 | 21.6 | 22.9 | 63.7 | 13.2 |
| PDX21 | JAK3, PTEN | positive (30%) | 30% | positive | positive (50%) | negative | 100.0 | 56.5 | 94.2 | 62.3 | 0.8 | 93.0 | 0.6 |

| 1. **Panel 2** | | **Neuropathological assessment** | | | | | **T/C [%]** | | | | | | | |
| --- | --- | --- | --- | --- | --- | --- | --- | --- | --- | --- | --- | --- | --- | --- |
| **Study number** | **Mutations** | **GFAP** | **Ki67/MIB2** | **MAP2** | **MGMT** | **IDH1 (R132H)** | **PBS (a)** | **TMZ (b)** | **Thalidomide (c)** | **TMZ/Thalidomide (d)** | **Everolimus (e)** | **TMZ/Everolimus (f)** | **Regorafenib (g)** | **TMZ/Regorafenib (h)** |
| PDX3 | FGFR, KDR | positive  (100%) | 40% | positive | negative | negative | 100.0 | 1.1 | 58.8 | 0.7 | 34.1 | 2.6 | 22.3 | 2.3 |
| PDX4 | EGFR, MET | positive (30%) | 40% | positive | negative | negative | 100.0 | 0.2 | 46.6 | 0.3 | 32.6 | 1.3 | 35.1 | 0.5 |
| PDX8 | APC, FLT3 | positive  (100%) | 10% | positive | negative | negative | 100.0 | 7.7 | 14.5 | 10.8 | 66.3 | 9.7 | 70.2 | 9.6 |
| PDX14 | KDR, KIT | positive (30%) | 90% | positive | positive (70%) | negative | 100.0 | 73.7 | 67.2 | 20.4 | 35.7 | 22.6 | 27.0 | 16.0 |
| PDX19 | no mutations | positive  (100%) | 85% | positive | negative | negative | 100.0 | 4.3 | 68.8 | 2.7 | 49.7 | 5.4 | 54.4 | 5.5 |
| PDX20 | KDR, PIK3CA | positive  (100%) | 80% | positive | negative | negative | 100.0 | 68.8 | 70.1 | 80.8 | 31.7 | 35.7 | 49.7 | 48.7 |

Table S2: Primary antibodies used for immunoblot analyses. Antibody dilutions for immunohistochemical analyses in parentheses.

|  | **Antibody** | **P- site** | **Company** | **Host** | **Dilution** | **kDa** | **Identifier** | **RRID** |
| --- | --- | --- | --- | --- | --- | --- | --- | --- |
| **PI3K/AKT/mTOR signaling** | p-4E-BP1 | Ser65 | Cell Signaling Technology | Rabbit | 1:1000 | 15-20 | #9456 | AB_823413 |
|  | 4E-BP1 | - | Cell Signaling Technology | Rabbit | 1:1000 | 15-20 | #9452 | AB_331692 |
|  | p-mTOR | Ser2448 | Cell Signaling Technology | Rabbit | 1:1000 | 289 | #5536 | AB_10691552 |
|  | mTOR (7C10) | - | Cell Signaling Technology | Rabbit | 1:1000 | 289 | #2983 | AB_2105622 |
|  | p-Raptor | Ser792 | Cell Signaling Technology | Rabbit | 1:1000 | 150 | #2083 | AB_2249475 |
|  | Raptor (24C12) | - | Cell Signaling Technology | Rabbit | 1:1000 | 150 | #2280 | AB_561245 |
|  | p-Rictor (D30A3) | Thr1135 | Cell Signaling Technology | Rabbit | 1:1000 | 200 | #3806S | AB_10557237 |
|  | Rictor | - | Cell Signaling Technology | Rabbit | 1:1000 | 200 | #2114 | AB_217996 |
|  | p-AKT | Ser473 | Cell Signaling Technology | Rabbit | 1:1000 | 60 | #4058 | AB_331168 |
|  | AKT | - | Cell Signaling Technology | Rabbit | 1:1000 | 60 | #9272 | AB_329827 |
|  | p-P70S6K | Thr421/Ser424 | Cell Signaling Technology | Rabbit | 1:1000 | 70,85 | #9204L | AB_2265913 |
|  | P70S6K | - | Cell Signaling Technology | Rabbit | 1:1000 | 70,85 | #9202 | AB_331676 |
|  | p-PTEN | Ser380 | Cell Signaling Technology | Rabbit | 1:1000 | 54 | #9551S | AB_331407 |
|  | PTEN | - | Cell Signaling Technology | Rabbit | 1:1000 | 54 | #9559 | AB_390810 |
|  | p-AMPKα | Thr172 | Cell Signaling Technology | Rabbit | 1:1000 | 62 | #2535 | AB_331250 |
|  | AMPKα (D63G4) | - | Cell Signaling Technology | Rabbit | 1:1000 | 62 | #5832 | AB_10624867 |
|  | p-P53 | Ser15 | Cell Signaling Technology | Mouse | 1:1000 | 53 | #9286 | AB_331741 |
|  | P53 (7F5) | - | Cell Signaling Technology | Rabbit | 1:1000 | 53 | #2527 | AB_331211 |
|  | MDM2 | - | Santa Cruz Biotechnology | Mouse | 1:1000 | 60,90 | sc-965 | AB_627920 |
| **eIF signaling** | eIF1A | - | Abcam | Rabbit | 1:1000 | 18 | Ab177939 | - |
|  | eIF2α (D7D3) | - | Cell Signaling Technology | Rabbit | 1:1000 | 38 | #5324 | AB_10692650 |
|  | p-eIF2α (D9G8) | Ser51 | Cell Signaling Technology | Rabbit | 1:1000 | 38 | #3398 | AB_2096481 |
|  | eIF3A | - | Cell Signaling Technology | Rabbit | 1:1000 | 166 | #2538 | AB_915861 |
|  | eIF3B (eIF3η D-9) | - | Santa Cruz Biotechnology | Mouse | 1:1000 | 116 | Sc-137215 | AB_2096734 |
|  | eIF3C | - | Cell Signaling Technology | Rabbit | 1:1000 | 110 | #2068 | AB_2096742 |
|  | eIF3D (eIF3ζ H-300) | - | Santa Cruz Biotechnology | Rabbit | 1:1000 | 64 | Sc-28856 | AB_2246358 |
|  | eIF3F | - | Abcam | Rabbit | 1:1000 | 38 | Ab74568 | AB_1523532 |
|  | eIF3H (D9C1) | - | Cell Signaling Technology | Rabbit | 1:1000 | 40 | #3413S | AB_2277726 |
|  | eIF3I (eIF3β A-8) | - | Santa Cruz Biotechnology | Mouse | 1:1000 | 36 | Sc-374155 | AB_10986412 |
|  | eIF3I | - | Sigma Aldrich | Rabbit | (1:500) | 36 | PA5-56301 | AB_2640966 |
|  | eIF3J | - | Cell Signaling Technology | Rabbit | 1:1000 | 35 | #3261S | AB_2097076 |
|  | eIF3K (2313C2a) | - | Santa Cruz Biotechnology | Mouse | 1:1000 | 25 | Sc-81262 | AB_2231026 |
|  | eIF3M (V-21) | - | Santa Cruz Biotechnology | Rabbit | 1:1000 | 43 | Sc-133541 | AB_10614157 |
|  | eIF4A | - | Cell Signaling Technology | Rabbit | 1:1000  (1:100) | 48 | #2490S | AB_10831840 |
|  | p-eIF4B | Ser406 | Cell Signaling Technology | Rabbit | 1:1000 | 80 | #5399S | AB_10695246 |
|  | eIF4B | - | Cell Signaling Technology | Rabbit | 1:1000 | 80 | #3592 | AB_2293388 |
|  | eIF4E | - | Cell Signaling Technology | Rabbit | 1:1000 | 25 | #9742 | AB_823488 |
|  | p-eIF4G | Ser1108 | Cell Signaling Technology | Rabbit | 1:1000 | 220 | #2441S | AB_2277632 |
|  | eIF4G | - | Cell Signaling Technology | Rabbit | 1:1000 | 220 | #2498 | AB_2096025 |
|  | eIF4H | - | Cell Signaling Technology | Rabbit | 1:1000  (1:40) | 27 | #3469S | AB_2096038 |
|  | eIF5 | - | GeneTex | Rabbit | 1:1000 | 49 | GTX114923 | AB_10730759 |
|  | eIF5A | - | Thermo Fisher Scientific | Rabbit | 1:1000 | 17 | PA5-29204 | AB_2546680 |
|  | eIF6 | - | GeneTex | Rabbit | 1:1000 | 26 | GTX63642 | - |
|  | eIF6 | - | Bethyl | Rabbit | (1:200) | 26 | A303 030A | AB_10754786 |
|  | GAPDH | - | Cell Signaling Technology | Rabbit | 1:2000 | 38 | #2118 | AB_561053 |

**Table S3: Drugs and dosages for chemosensitivity testings in GBM PDX.** Agent, trade name, company dosage and application (administration and frequency) used for treatment panel 1 (single treatment) and treatment panel 2 (combination treatment) are listed.

| ***Treatment panel 1 (single treatment)*** | | | |
| --- | --- | --- | --- |
| **Agent** | **Company** | **Dosage** | **Application** |
| Everolimus | Novartis, Basel, Switzerland | 5 mg/kg | orally, (days 1–5)x2 |
| Sorafenib | Bayer, Leverkusen, Germany | 80 mg/kg | orally, (days 1–5)x2 |
| Bevacizumab | Roche, Basel, Switzerland | 10 mg/kg | Intraperitoneally, (three times a week)x2 |
| Irinotecan | Fresenius, Bad Homburg, Germany | 15 mg/kg | intraperitoneally, days 1–5 |
| Salinomycin | Sigma-Aldrich, St.Louis, MI, US | 10 mg/kg | orally, days 1–14 |
| Temozolomide | MSD, Kenilworth, NJ, US | 90 mg/kg | orally, days 1–5 |
| ***Treatment panel 2 (combination treatment)*** | | | |
| **Agent** | **Company** | **Dosage** | **Application** |
| Temozolomide | MSD, Kenilworth, NJ, US | 25 mg/kg | orally, (days 1–5)x3 |
| Thalidomide | Caesar & Loretz GmbH, Hilden, Germany | 400 mg/kg | orally, (days 1–7)x3 |
| Temozolomide/thalidomide |  | dosage equal to the single treatment | |
| Everolimus | Novartis, Basel, Switzerland | 5 mg/kg | orally, (days 1–5)x3 |
| Temozolomide/everolimus |  | dosage equal to the single treatment | |
| Regorafenib | Bayer, Leverkusen, Germany | 10 mg/kg | orally, (days 1–7)x3; |
| Temozolomide/regorafenib |  | dosage equal to the single treatment | |

**Key Resources Table**

| REAGENT RESOURCE | SOURCE | | IDENTIFIER | |
| --- | --- | --- | --- | --- |
| **Antibodies** | | | | |
| Primary antibodies | This paper | | see Table S6 | |
| Sec. anti-rabbit; hrp conj. | GE Healthcare | | Cat# NA934-1ML; RRID:AB_772206 | |
| Sec. Anti-mouse; hrp conj. | GE Healthcare | | NXA931-1ML; RRID: AB_772209 | |
| **Biological Samples** | | | | |
| Brain tumour tissue | Institute of Pathology, Medical University of Graz | | In this study | |
| Explanted tumour tissue from patient-derived xenografts (PDX) | EPO- Experimental Pharmacology  & Oncology Berlin-Buch GmbH | | In this study | |
| **Chemicals, Peptides, and Recombinant Proteins** | | | | |
| Biorad Protein Assay Dye Reagent | Bio-Rad Laboratories | | Cat# 500-0006 | |
| Amersham ECL Prime Western blotting detection Reagent | GE Healthcare | | RPN 2236 | |
| Amersham ECL Select Western blotting detection Reagent | GE Healthcare | | RPN 2235 | |
| cOmplete™ Protease Inhibitor Cocktail | Roche | | Cat# 04693116001 | |
| PhosSTOP™ phosphatase inhibitor | Roche | | Cat# 4906845001 | |
| ^Bevacizumab (Avastin®)^ | Roche | | Cat# QS51031; CAS ID:216974-75-3 | |
| Everolimus (Certican^®^) | Novartis | | CAS ID: 159351-69-6 | |
| Irinotecan | Fresenius Kabi | | CAS ID: 97682-44-5 | |
| Regorafenib (Stivarga^®^) | Bayer | | CAS ID: 755037-03-7 | |
| Salinomycin | Sigma-Aldrich | | Cat# S4526;  CAS ID: 53003-10-4 | |
| Sorafenib (Nexavar^®^) | Bayer | | CAS ID: 284461-73-0 | |
| Temozolomide (Temodal^®^) | Merck | | CAS ID: 85622-93-1 | |
| Thalidomide | Caesar & Loretz GmbH | | CAS ID: 50-35-1 | |
| **Critical Commercial Assays** | | | | |
| UltraView DAB Detection-Kit | | Ventana | | Cat# 760-500 |
| QIAamp DNA Mini Kit | | Qiagen | | Cat# 51304 |
| TruSeq Amplicon-Cancer Panel | | Illumina | | Cat# FC-130-1008 |
| **Experimental Models: Organisms/Strains** | | | | |
| Mouse: NOD/SCID | Taconic, Lille Skensved, Denmark | | NOD/MrkBomTac-Prkdc^scid^; RRID: IMSR_TAC:nodsc | |
| Mouse: NMRI:nu/nu | Janvier Labs, Le Genest-Saint-Isle, France | | Rj:NMRI-Foxn1^nu/nu^ | |
| **Software and Algorithms** | | | | |
| ImageJ | (19 Schneider et al., 2012) | | <https://imagej.nih.gov/ij/> | |
| Graphpad PRISM version 4.03 | GraphPad software Inc. | | <https://www.graphpad.com/> | |
| R version 3.3.0 | R software | | [www.r-project.org](http://www.r-project.org/) | |
| Harrell FE Jr. Package ‘HMISC’ used version “update June 4 2010” | Frank E Harrell Jr., April 6 2006 (update: June 4 2010) | | <https://cran.r-project.org/web/packages/Hmisc/index.html> | |

| **Table S4:** Classification of response rate to treatment according to tumour growth inhibition ratio (T/C %). | |
| --- | --- |
| **Responserate rete** | **T/C** |
| Non-responder | >50% |
| Minimal responder | 50%-35% |
| Weak responder | 35%-20% |
| Moderate responder | 20%-5% |
| Strong responder | <5% |

| **Table S5:** Spearman correlation analysis between tumour growth inhibition ratio (T/C %) and various eIFs (CC: Correlation coefficient). | | | | | | | | | | | | | | | | | | | |  |  |  |
| --- | --- | --- | --- | --- | --- | --- | --- | --- | --- | --- | --- | --- | --- | --- | --- | --- | --- | --- | --- | --- | --- | --- |
|  |  | eIF1A | eIF2α | p-eIF2α | eIF3A | eIF3B | eIF3C | eIF3d | eIF3M | eIF3H | eIF3I | eIF3J | eIF3K | eIF4a | eIF4B | P-eIF4B | eIF4E | eIF4H | eIF4G | | eIF5 | eIF6 |
| T/C% | CC | -,160 | -,471 | -,402 | -,205 | -,274 | -,171 | -,395 | -,341 | -,487 | **-,593^*^** | -,346 | ,100 | -,114 | -,182 | -,289 | -,272 | -,185 | ,267 | | -,181 | -,454 |
|  | P-value | ,555 | ,065 | ,123 | ,483 | ,305 | ,558 | ,130 | ,196 | ,056 | **,015** | ,189 | ,714 | ,675 | ,615 | ,277 | ,308 | ,493 | ,317 | | ,573 | ,077 |
|  | N | 16 | 16 | 16 | 14 | 16 | 14 | 16 | 16 | 16 | 16 | 16 | 16 | 16 | 10 | 16 | 16 | 16 | 16 | | 12 | 16 |
